# Clinical Factors Associated with Time-Specific Distribution of 18F-Fluorodeoxyglucose in Large-Vessel Vasculitis

**DOI:** 10.1038/s41598-019-51800-x

**Published:** 2019-10-23

**Authors:** Joel S. Rosenblum, Kaitlin A. Quinn, Casey A. Rimland, Nehal N. Mehta, Mark A. Ahlman, Peter C. Grayson

**Affiliations:** 1 Systemic Autoimmunity Branch, NIAMS, Bethesda, Maryland USA; 2 Division of Rheumatology, MedStar Georgetown University Hospital, Washington, District of Columbia USA; 3 University of North Carolina at Chapel Hill School of Medicine, Medical Scientist Training Program, Chapel Hill, NC USA; 4 Cardiovascular Branch, National Heart, Lung, and Blood Institute, Bethesda, Maryland USA; 5 Radiology and Imaging Sciences, National Institutes of Health Clinical Center, Bethesda, Maryland USA

**Keywords:** Outcomes research, Vasculitis syndromes

## Abstract

^18^F-fluorodeoxyglucose (FDG) positron emission tomography (PET) can detect vascular inflammation in large-vessel vasculitis (LVV). Clinical factors that influence distribution of FDG into the arterial wall and other tissues have not been characterized in LVV. Understanding these factors will inform analytic strategies to quantify vascular PET activity. Patients with LVV (n = 69) underwent 141 paired FDG-PET imaging studies at one and two hours per a delayed image acquisition protocol. Arterial uptake was quantified as standardized uptake values (SUV_Max_). SUV_Mean_ values were obtained for background tissues (blood pool, liver, spleen). Target-to-background ratios (TBRs) were calculated for each background tissue. Mixed model multivariable linear regression was used to identify time-dependent associations between FDG uptake and selected clinical features. Clinical factors associated with FDG distribution differed in a tissue- and time-dependent manner. Age, body mass index, and C-reactive protein were significantly associated with arterial FDG uptake at both time points. Clearance factors (e.g. glomerular filtration rate) were significantly associated with FDG uptake in background tissues at one hour but were weakly or not associated at two hours. TBRs using liver or blood pool at two hours were most strongly associated with vasculitis-related factors. These findings inform standardization of FDG-PET protocols and analytic approaches in LVV.

## Introduction

Giant cell arteritis (GCA) and Takayasu’s arteritis (TAK) are the two major forms of large-vessel vasculitis (LVV) defined by immune-mediated inflammation of the large arteries leading to stenoses, occlusions, and aneurysms^1–3^. FDG-PET is useful to detect and potentially monitor treatment response in LVV^4–7^. Currently, most studies in LVV use qualitative analytical approaches to measure arterial FDG uptake which depends on the expertise of the reader and may lack precision due to categorized resultant metrics (e.g. visual FDG uptake in the artery relative to the liver). In comparison, semiquantitative analysis may be preferred to limit the effect of reader bias, increase reproducibility, and optimize precision. To date, few studies have used semiquantitative analysis to assess the utility of FDG-PET in LVV^8,9^. These studies employed a variety of approaches, including direct measurement of arterial FDG uptake^10^ and measurement of arterial FDG uptake normalized to a background tissue using target-to-background ratios (TBR)^11,12^. Moreover, different background tissues (e.g. liver^13^, blood pool^11,14^) have been used to normalize FDG uptake and image acquisition time can vary from one study to the next, making study comparisons difficult.

Recent consensus guidelines on the role of FDG-PET in LVV offered few evidence-based recommendations about the use of semiquantitative analysis to measure vascular FDG uptake^8^. Most evidence supporting these recommendations was based on studies in atherosclerosis rather than LVV^8,15^. Recommendations included normalization of arterial FDG uptake to blood pool and acquisition of images at a minimum of 60 minutes after radiotracer injection. In studies of atherosclerosis, delayed imaging (i.e. 90 minutes or more) may be advantageous to allow for more FDG uptake into the arterial wall and clearance from the blood pool^16–18^. Ultimately the guidelines indicated that further research is needed to inform recommendations on semiquantitative analytic approaches in LVV.

Previous studies have shown that various factors can influence the distribution of FDG into specific tissues, including the arterial wall and tissues used for normalization of arterial FDG uptake^8,19^. The clinical factors associated with FDG distribution into the arterial wall and background tissues have not been comprehensively assessed in LVV. Understanding to what extent factors related and not related to vasculitis are associated with arterial FDG uptake may provide insight into the underlying pathophysiology of vascular PET activity and will inform the development of PET-based outcome measures of vascular inflammation. This study employed a unique design; whereby, sequential FDG-PET images were obtained in the same patients one and two hours after a single injection of FDG. This sequential imaging protocol enabled evaluation of time-dependent factors associated with FDG distribution in patients with LVV.

## Results

### Patient characteristics

A total of 69 patients (TAK = 31 patients; GCA = 38 patients) were recruited into the study. A total of 141 paired one-hour and two-hour FDG-PET scans were included (TAK = 51 studies; GCA = 90 studies). Baseline demographics of the study population are shown in Table 1. Patients with TAK were notably younger than patients with GCA (35.6 vs 68.4 years, p < 0.01), consistent with expected demographic differences between these diseases.

**Table 1:** Study Population Baseline Characteristics.

Variable	Total N = 69	TAK N = 31	GCA N = 38
Number of Patients with Multiple Study Visits (2 scans per visit)			
1 Study Visit	36	19	17
2 Study Visits	10	5	5
≥3 Study Visits	23	7	16
Age (years ± SD)	53.17 ± 19.8	35.55 ± 11.9	68.37 ± 8.6
Sex (female, %)	52 (75.3)	22 (71.0)	30(78.9)
CRP (mg/L ± SD)	10.62 ± 21.2	13.34 ± 22.2	8.4 ± 20.4
Total WBC (10^9^/L ± SD)	9.48 ± 3.3	9.92 ± 3.9	9.11 ± 2.7
Fasting Glucose (mg/dL ± SD)	86.31 ± 15.6	86.06 ± 13.0	86.53 ± 17.6
Prednisone (mg/day ± SD)	12.16 ± 16.7	9.40 ± 13.7	14.41 ± 18.7
Other Immunosuppressant Therapy (%)	36 (52.2)	20(64.5)	16(42.1)
One-Hour UT (minutes ± SD)	71.26 ± 21.9	75.1 ± 31.6	68.12 ± 7.0
Two-Hour UT (minutes ± SD)	125.55±17.7	123.35 ± 24.9	127.34 ± 8.0
BMI (±SD)	25.54 ± 4.8	25.31±5.4	25.74 ± 4.2
GFR (mL/min per 1.73 m^2^ ± SD)	85.36 ± 23.1	98.29 ± 23.9	74.82 ± 16.2
Hematocrit (% ± SD)	39.51 ± 4.3	38.9 ± 3.9	40.02 ± 4.6
Physician Global (range 0–10) (±SD)	1.39 ± 1.9	1.26 ± 1.8	1.5 ± 1.9

GCA, giant cell arteritis; TAK, Takayasu’s arteritis; CRP, C-Reactive Protein; Total WBC, Total White Blood Cell Count; UT, uptake time; BMI, Body Mass Index; GFR, Glomerular Filtration Rate. Age, CRP, Total WBC, Fasting Glucose, Prednisone, One-hour and Two-hour Image Acquisition Time, BMI, GFR, Hematocrit, and Physician Global are all reported as mean ± standard deviation. Sex and Other Immunosuppressant Therapy are reported as number of individuals (percentage).

### Inter- and intra-reader reliability

Inter- and intra-rater reliability was excellent for contouring of the arterial wall (inter-rater r = 0.99, p < 0.01; intra-rater r = 0.99, p < 0.01) and all background tissues: blood pool (inter-rater r = 0.97, p < 0.01; intra-rater r = 0.98, p < 0.01), liver (inter-rater r = 0.99, p < 0.01; intra-rater r = 0.99, p < 0.01), and spleen (inter-rater r = 0.94, p < 0.0; intra-rater r = 0.97, p < 0.01) (Supplementary Figs 1 and 2).

### Changes in FDG uptake at one and two-hour imaging acquisition times

FDG uptake in the artery, liver, blood pool, and spleen were each significantly different (p < 0.01) between the one and two-hour imaging times as shown in Table 2. FDG uptake consistently increased in solid tissues and decreased in the blood pool over time. The percentage of change of FDG uptake from one to two hours was greatest for the artery, where the percentage of FDG uptake increased by 40.5% (mean ± SD; one-hour = 2.34 ± 0.4, two-hour = 3.29 ± 1.0, p < 0.01), followed by spleen then liver. In contrast, blood pool FDG activity was greater at one-hour and decreased by 9.6% at the two-hour imaging acquisition time (mean ± SD; one-hour = 1.49 ± 0.2, two-hour = 1.36 ± 0.2, p < 0.01). There was an increase in the CV from the one-hour to two-hour acquisition times in all tissues. Among background tissue, the lowest CV was observed in blood pool (CV one-hour = 0.13; CV two-hour = 0.15).

**Table 2:** Standardized Uptake Values at One and Two-Hour Imaging Time Points.

Tissue Type	One-Hour PET Mean ± SD (CV)	Two-Hour PET Mean ± SD (CV)	Percent Change in FDG Uptake (P Value)
Artery	2.34 ± 0.4 (0.17)	3.29 ± 1.0 (0.30)	+40.5% (<0.01)
Liver	2.24 ± 0.3 (0.13)	2.42 ± 0.4 (0.17)	+8.0% (<0.01)
Blood Pool	1.49 ± 0.2 (0.13)	1.36 ± 0.2 (0.15)	−9.6% (<0.01)
Spleen	1.75 ± 0.3 (0.17)	2.15 ± 0.5 (0.23	+22.9% (<0.01)

Values are presented as mean standardized uptake values ± standard deviation (SD).

CV, Coefficient of variation. Percent change was calculated as the percent increase or decrease of FDG uptake measured by SUV_Max_ in artery and SUV_Mean_ in background tissues from one-hour to two-hour imaging.

### FDG uptake in specific background tissues

#### FDG uptake in blood pool

BMI and UT were associated with FDG distribution in the blood pool at both time points (one-hour: BMI, B = 0.01, p < 0.01; UT, B = −0.005, p < 0.01) (two-hour: BMI, B = 0.01, p < 0.01; UT, B = −0.003, p < 0.01). Additionally, GFR was negatively associated with FDG distribution in the blood pool at one-hour imaging (GFR, B = −0.003, p = 0.01), but this association did not persist during two-hour imaging (GFR, B = −0.0005, p = 0.63). Increased hematocrit was associated with decreased FDG uptake in the blood pool at one-hour imaging (B = −0.01, p = 0.01), while age (B = 0.005, p < 0.01) and female sex (B = 0.10, p = 0.02) were positively associated with FDG uptake in the blood pool only during two-hour imaging. Apart from hematocrit during one-hour imaging there were no other vasculitis-related variables associated with FDG uptake in blood pool. Normalized beta-estimates indicated the three factors most strongly associated with FDG uptake in the blood pool were UT, GFR, and BMI at one-hour and age, BMI, and sex at two-hour imaging (Supplementary Table 2).

#### FDG uptake in liver

UT was negatively associated with FDG distribution to the liver during one-hour imaging (B = −0.003, p < 0.01). Although not statistically significant, patients with higher GFR had decreased FDG uptake in the liver at one-hour (B = −0.003, p = 0.06). These associations did not persist during two-hour imaging (UT, B = 0.0002, p = 0.90; GFR, B = −0.0001, p = 0.95). Although no vasculitis-related variables were significantly associated with FDG uptake at either time point, patients taking immunosuppressant medications had on average greater FDG uptake in the liver during one-hour imaging (B = 0.08, p = 0.09). Normalized beta-estimates indicated the three factors most strongly associated with FDG uptake in the liver were BMI, UT, and GFR at one-hour and BMI, age, and sex at two-hour imaging (Supplementary Table 3).

#### FDG uptake in spleen

FDG uptake in the spleen was associated with BMI during both one-hour (BMI, B = 0.02, p < 0.01) and two-hour imaging (BMI, B = 0.05, p < 0.01). While not significant, there was a negative association with PGA (B = −0.03, p = 0.07) and a positive association with CRP (B = 0.002, p = 0.07) observed during one-hour imaging. Normalized beta-estimates indicated the three factors most strongly associated with FDG uptake in the spleen were BMI, PGA, and hematocrit at one-hour and BMI, age, and Total WBC at two-hour imaging (Supplementary Table 4).

### FDG uptake in the artery

#### SUV measurement of arterial FDG uptake without background normalization

Increased age, CRP, and BMI were independently associated with arterial FDG uptake during one-hour (age, B = 0.01, p < 0.01; CRP, B = 0.007, p < 0.01; BMI, B = 0.03, p < 0.01) and two-hour imaging (age, B = 0.03, p < 0.01; CRP, B = 0.01, p < 0.01; BMI, B = 0.05, p = 0.02). Fasting glucose level was negatively associated with arterial FDG uptake only during two-hour imaging (B = −0.01, p = 0.045). Treatment with immunosuppressant medications was associated with decreased arterial FDG uptake during two-hour (B = −0.37, p = 0.02) but not one-hour imaging (B = −0.15, p = 0.14). Clinical assessment of disease activity was not associated with arterial FDG uptake at either time point (PGA, one-hour B = 0.004, p = 0.83; two-hour B = 0.04, p = 0.48). Complete results from the mixed multivariable linear regression models at both time points are shown in Table 3. Normalized beta-estimates indicated the factors most strongly associated with FDG uptake in the arterial wall were age, BMI, and CRP at both time points (Table 3). In a separate regression model, type of large-vessel vasculitis (GCA versus TAK) was included as a predictor variable but was not significantly associated with arterial PET activity, supporting the appropriateness of studying these diseases in composite (data not shown).

**Table 3:** Clinical Factors Associated with Arterial FDG Uptake at One and Two-Hour Imaging Time Points.

Variable	One-Hour			Two-Hour		
	B Estimate	β Estimate	P-value	B Estimate	β Estimate	P-value
Sex (female)	−0.07	0.08	0.47	0.13	0.05	0.63
Age (years)	0.01	0.48	<0.01	0.03	0.47	<0.01
PGA(0–10)	0.004	0.02	0.83	0.04	0.06	0.48
CRP (mg/L)	0.007	0.3	<0.01	0.01	0.22	<0.01
Total WBC (10^9^/L)	−0.0007	0.05	0.81	0.02	0.05	0.55
Fasting Glucose (mg/dL)	−0.002	0.12	0.22	−0.01	0.16	0.045
GFR (mL/min per 1.73 m^2^)	0.0005	0.03	0.80	0.003	0.06	0.62
Hematocrit (%)	−0.01	0.1	0.26	−0.01	0.05	0.60
Immune Medications (yes)	−0.15	0.08	0.14	−0.37	0.17	0.02
Prednisone (mg/day)	−0.001	0.06	0.21	−0.003	0.04	0.58
UT (per minute)	−0.001	0.06	0.33	−0.0001	0.002	0.98
BMI	0.03	0.4	<0.01	0.05	0.24	0.02

BMI, Body Mass Index; CRP, C-Reactive Protein; GFR, Glomerular Filtration Rate; Immune Medications (yes), taking immunosuppressant medications other than glucocorticoids; PGA, Physician Global Assessment; Total WBC, Total White Blood Cell Count. Normalized Beta Estimates (β Estimate) given as absolute values; Beta Estimates (B Estimate).

#### Assessment of arterial FDG uptake normalized to background tissues

The clinical factors that were associated with arterial FDG uptake were different when arterial FDG uptake was normalized to background tissue. Age remained associated with arterial FDG uptake normalized to each background (B = 0.004–0.01, p < 0.01) except for TBR_Blood pool_ at two hours, but the strength of association was reduced compared to the association of age and arterial FDG uptake without normalization (B = 0.01–0.03, p < 0.01). CRP also remained associated with all TBRs, with the exception of TBR_Spleen_ during two-hour imaging. While SUV_Sum_ was not associated with any imaging-related factors during one-hour imaging, TBR_Blood pool_ was significantly associated with UT (B = 0.005, p < 0.01) and GFR (B = −0.003, p = 0.01) at that time point. Use of immunosuppressant medication was negatively associated with TBR_Liver_ (B = −0.07, p = 0.03) during one-hour time imaging. Complete results from the mixed multivariable linear regression models for all TBRs are shown in Supplementary Tables 5–7. Normalized beta estimates indicated the three factors most strongly associated with TBR_Blood pool_ were age, CRP, and UT at one-hour and CRP, age, and fasting glucose at two-hour imaging (Supplementary Table 5). The three factors most strongly associated with TBR_Liver_ were age, CRP, and GFR at one-hour and CRP, age, and fasting glucose at two-hour imaging (Supplementary Table 6). The three factors most strongly associated with TBR_Spleen_ were age, CRP, and total WBC at one-hour and CRP, age, and GFR at two-hour imaging (Supplementary Table 7).

A summary of all significant factors in each of the mixed multivariable linear regression model is shown in Table 4.

**Table 4:** Summary of Clinical Factors Associated with FDG Distribution at One and Two-Hour Imaging Time Points.

Tissue	One-Hour PET	Two-Hour PET
Artery	Age (β = 0.01, p < 0.01) CRP (β = 0.007, p < 0.01) BMI (β = 0.03, p < 0.01)	Age (β = 0.02, p < 0.01) CRP (β = 0.01, p < 0.01) Fasting Glc (β = −0.01, p = 0.04) Immune Meds (β = −0.37, p = 0.02) BMI (β = 0.05, p = 0.02)
Liver	UT (β = −0.003, p < 0.01) BMI (β = 0.02, p < 0.01)	Age (β = 0.006, p = 0.02) BMI (β = 0.03, p < 0.01)
Blood Pool	GFR (β = −0.003, p = 0.01) UT (β = −0.005, p < 0.01) Hematocrit (β = −0.01, p = 0.01) BMI (β = 0.01, p < 0.01)	Age (β = 0.005, p < 0.01) UT (β = −0.003, p < 0.01) BMI (β = 0.01, p < 0.01)
Spleen	BMI (β = 0.02, p < 0.01)	BMI (β = 0.05, p < 0.01)
TBR Liver	Age (β = 0.004, p < 0.01) CRP (β = 0.004, p < 0.01) Immune Meds (β = −0.07, p = 0.03)	Age (β = 0.008, p = 0.02) CRP (β = 0.01, p < 0.01) Fasting Glc (β = −0.005, p = 0.046)
TBR Blood Pool	Age (β = 0.007, p < 0.01) CRP (β = 0.004, p = 0.03) GFR (β = 0.004, p = 0.04) UT (β = 0.005, p < 0.01)	CRP (β = 0.02, p < 0.01) Fasting Glc (β = −0.001, p = 0.02)
TBR Spleen	Age (β = 0.006, p < 0.01) CRP (β = 0.003, p = 0.045)	Age (β = 0.009, p = 0.02)

*The following variables were included in each regression model: sex (female), body mass index (BMI), age in years, C-reactive protein (CRP, mg/L), total white blood cell count (WBC, 10^9^/L), treatment with immunosuppressant medications other than glucocorticoids (Immune Meds, Yes vs No), daily prednisone dose (mg), physician global assessment (PGA, 0–10 scale), hematocrit (%), uptake time (UT, minutes from injection of FDG), glomerular filtration rate (GFR, mL/min per 1.73 m^2^), fasting glucose (Fasting Glc, mg/dL). TBR, target-to-background ratio with artery target tissue.

## Discussion

Quantification of FDG uptake in the arterial wall can be affected by many factors. Previous studies in atherosclerosis have examined how multiple clinical factors influence FDG distribution in the arterial wall and other tissues^19,20^. This study is unique in that factors associated with FDG distribution were studied in a time-dependent and tissue-specific manner in a cohort of patients with LVV. Consistent with prior reports, delayed imaging from one to two hours was associated with increased FDG uptake in the arterial wall and other tissues, with decreased FDG uptake in the blood pool^8,14^. Clinical factors and strength of association with FDG uptake in the artery and background tissues differed during one-hour versus and two-hour imaging and were dependent on whether SUV metrics or TBRs were used to quantify FDG uptake. While the objective of this study was not to derive an optimal semiquantitative metric to diagnose or monitor vascular PET activity in LVV, this work should inform the future development of PET-based outcome measures by providing a deeper understanding of the strengths and limitations of different TBRs at specific imaging time points.

This study provides evidence in support of delayed image acquisition in LVV. Delayed image acquisition influences subjective interpretation of FDG-PET scans in vasculitis, as scans performed at later time points are more likely to be interpreted as active vasculitis^21^. Our findings align with data from previous studies in atherosclerosis that show delayed imaging increases the sensitivity to detect FDG uptake in the arterial wall^22,23^. In LVV, delayed imaging increases the vascular-to-blood pool ratio of FDG. The average uptake of FDG increased by 40.5% in the arterial wall between one-hour to two-hour imaging, while FDG uptake in the blood pool decreased by 9.6% over a similar time frame. Additionally, time-dependent associations between arterial FDG uptake and clinical factors related and unrelated to vasculitis were identified. At the later time point, CRP levels were more strongly associated with arterial FDG uptake, indicating that delayed imaging may increase the sensitivity to detect vascular inflammation. However, significant associations between arterial FDG uptake and factors not directly related to vasculitis, such as BMI and age, were also stronger at the two-hour compared to one-hour time point.

Interestingly, there were few vasculitis related factors associated with arterial FDG uptake. These findings are consistent with cross-sectional analyses that demonstrate weak differences in FDG uptake between patients with LVV and controls by semiquantitative analysis^11^. Most patients included in the cohort were studied later in the course of disease rather than at diagnosis. This may explain why clinical assessment of disease activity was not associated with arterial FDG uptake, as a significant burden of vascular PET activity has been observed during periods of clinical remission in patients with LVV^5,24^. Similarly, many patients in the cohort were taking glucocorticoids or other immunosuppressant medications, which likely reduced the overall burden of vascular PET activity^25^. Given the relatively modest sample sizes, use of immunosuppressant medications was studied as a composite variable, and studying the effect of specific medications may be more informative. Similarly, cumulative glucocorticoid exposure in the immediate period prior to imaging rather than daily prednisone dose may be better linked to FDG activity. Ultimately, quantification of arterial FDG uptake may be a better biomarker of vascular inflammation in longitudinal studies that assess the effect of a given treatment on individual patients rather than across populations.

Understanding the time-dependent factors associated with FDG distribution in arteries and background tissues may enable investigators to account for potential confounding variables when developing outcome measures for arterial FDG uptake. Age, in particular, was strongly associated with vascular FDG uptake, consistent with prior studies^19^. Potential differences in age and BMI could bias studies that compare arterial SUVs if these factors are imbalanced between study groups. Using TBRs can lessen the impact of BMI but may introduce other potential confounding variables. This is illustrated by the potential impact of clearance-related factors on TBR_Blood pool_ during one-hour imaging. UT and GFR were not associated with FDG uptake in the artery at one hour but were associated with FDG uptake in blood pool. When arterial uptake was normalized to blood pool at one-hour imaging, clearance-related factors were strongly associated with the resultant TBR metric due to associations with background rather than target tissue. At two hours, GFR was no longer associated with blood pool activity and the strength of association with UT was markedly reduced. Consequently, clearance-related factors were no longer associated with the resultant TBR_Blood Pool_ metric at two-hour time imaging. Recent guidelines on FDG-PET imaging in LVV have recommended a minimum of 60 minutes between intravenous FDG injection and image acquisition and have recommended the use of TBR, normalizing to venous blood pool, instead of SUV for the quantification of arterial wall FDG uptake^8^. Findings from this study show that normalization of arterial wall uptake to venous blood pool activity may provide a good reference for assessing vascular imaging; however, delayed two-hour imaging would be preferable to reduce the potential impact of clearance-related factors. FDG-PET-based imaging studies that use one-hour image acquisition should be mindful of potential imbalances of clearance-related factors between study groups that could bias results.

Findings from this study suggest that imaging time point, rather than a specific background tissue, is more important for studying normalized arterial FDG uptake. At one hour, there were many potential confounding variables associated with FDG uptake in each of the background tissues. At two hours, there were fewer associated potential confounding variables and no vasculitis-related variables significantly associated with FDG uptake in any of those tissues. Vasculitis-related variables were only associated with TBR_Liver_ or TBR_Blood pool_ and not TBR_Spleen_. Further, the highest coefficient of variation of FDG uptake was observed in spleen compared to liver and blood pool. For these reasons, liver and blood pool may be preferable over spleen as a background tissue; however, future longitudinal studies of vascular PET activity in LVV should further explore the utility of different background tissues, being mindful of tissue-specific and time-dependent potential confounding variables.

This study has several unique strengths. Serial imaging by FDG-PET at two time-points on the same day enabled identification of time-dependent clinical factors associated with FDG distribution. All patients underwent a standardized imaging protocol using the same set of scanners with a centralized reader to generate semiquantitative metrics of FDG uptake per a specific contouring approach. Reliability analyses demonstrated excellent reproducibility of the imaging contouring procedure. Since arterial FDG distribution in LVV is typically diffuse throughout the large arteries^26,27^, a global summary score of quantitative vascular PET uptake throughout the aorta and branch arteries was used as an outcome measure to study the clinical factors associated with arterial FDG uptake in LVV. This summation approach parallels qualitative summary scores previously employed by our group and others to measure global arterial PET activity^5,28^. Imaging studies were performed prospectively in all patients within the cohort, minimizing the selection bias issues commonly encountered in retrospective studies of FDG-PET in LVV.

There are several potential study limitations to consider. Patients underwent serial imaging on two different imaging platforms: PET-MR at one-hour and PET-CT at two-hours. Scanner specific differences will likely contribute to differences in the strength of associations observed at the different imaging time points. Although Takayasu’s arteritis can affect young populations, children were excluded from multiple PET acquisitions on the same day to minimize radiation exposure. Moreover, this study does not provide information about how vascular imaging should be used in clinical practice, where potential risks, such as repetitive radiation exposure in younger populations, must be considered. While this study identified many time-dependent factors associated with FDG distribution into tissues, unmeasured confounders likely exist that also impact the distribution of FDG in patients with LVV. Specifically, limited information was recorded about atherosclerosis-related risk variables including lipid levels or information related to cardiovascular risk modification. Finally, while this study employed TBR calculations to quantify vascular FDG uptake, alternative analytic methods, such as measurement of total lesion glycolysis could be considered.

In conclusion, this study highlights the complexities of semiquantitative assessment of arterial PET activity in LVV and provides data that can inform standardization of imaging acquisition protocols and analytical approaches in LVV. Delayed imaging at two hours compared to one hour appears to be preferable in vascular FDG-PET imaging to allow more time for FDG to distribute into the arterial wall and clear from the blood pool. Specific factors are associated with the distribution of FDG in a tissue-specific time-dependent manner. Carefully designed studies that minimize imbalances of these factors are required to compare arterial FDG uptake among different populations. Normalization of arterial FDG uptake to a background tissue is advantageous to minimize the potential effects of body weight and radiotracer injection dose from one study to the next^8,17^; however, TBR outcome measures are susceptible to confounding effects of factors that are associated with FDG distribution in both the artery and the background tissue. While FDG-PET may be useful to monitor vascular inflammation in LVV, the development and validation of disease-specific semiquantitative metrics of vascular PET activity is prerequisite to unlock the utility of imaging-based outcome measures in these diseases.

## Methods

### Study population

Patients ≥18 years of age with LVV were recruited into a prospective, observational cohort at the National Institutes of Health (NIH) in Bethesda, Maryland, USA. All patients with LVV fulfilled the 1990 American College of Rheumatology (ACR) Classification Criteria for Takayasu’s arteritis^29^ or modified 1990 ACR Criteria for giant cell arteritis^30,31^. Patients could be enrolled at any point in the disease course.

### Visit schedule

All participants underwent clinical and laboratory assessments at the NIH clinical center within 24 hours of imaging acquisition. When feasible, patients underwent repeat assessments at 6-month intervals.

### Clinical and laboratory assessments

All clinical and laboratory assessments were collected blinded to imaging results. Clinical and laboratory assessment factors were divided into three groups; demographic, vasculitis-related, imaging-related. Demographic variables included body mass index (BMI), age, and sex. Vasculitis-related variables included clinical assessment of disease activity, c-reactive protein (CRP), total white blood cell count (WBC), hematocrit, daily prednisone dose, and use of additional immunosuppressive medications other than glucocorticoids (yes vs no). As there are no validated disease activity indices for both TAK and GCA, clinical assessment of disease activity was measured using the Physician Global Assessment (PGA) scale which was scored from 0 (remission) to 10 (severe disease). Imaging-related variables included glomerular filtration rate (GFR)^32^, exact time in minutes from radiotracer injection to image acquisition (uptake time, UT), and fasting glucose at the time of injection.

### FDG-PET imaging protocols

Patients were instructed to consume a carbohydrate-sparse meal the day prior to imaging, and fast on the day of imaging. Patients with a GFR lower than 30 mL/min per 1.73 m^2^ and/or a fasting glucose above 7.1 mmol/L were excluded from the study. All patients received a single, fixed dose of 10 mCi FDG.

Patients underwent simultaneous PET-MR (3 T Biograph mMR - Siemens Medical Solutions, Erlangen, Germany) approximately one hour after injection per a standardized imaging protocol: axial 2D free breathing, ECG-gated turbo spin echo double inversion recovery pulse sequence (TSE-DIR) with spectral attenuated inversion recovery (SPAIR). Acquisition parameters included automatically selected (Ti) by ECG, two R-to-R intervals employed resulting in repetition times (Tr) between 1,000–1,300 mSec. The echo time (Te) was set to 27 mSec. Slice thickness was set to 5 mm, matrix was 187 × 384 with a receiver bandwidth (Rbw) of 766 hertz per pixel (hz/px), flip angle was set to 160 degrees, and the echo-train length (ETL) was set to 11. Parallel imaging (GRAPPA-Generalized Auto-calibrating Partial Parallel Acquisition) was used to reduce overall scan time by a factor of 2. 2D axial TSE-STIR (turbo-spin echo short tau inversion recovery) images were obtained. The Ti was 220 mSec, Tr 4,500 mSec, and Te was 73 mSec. Slice thickness was set to 8 mm, matrix 284 × 448, and Rbw of 302 hz/pz, flip angle 120 degrees, and ETL was set to 11. GRAPPA parallel imaging was also used. Reconstruction of PET-MR images employed MR-based attenuation correction and iterative reconstruction (3 iterations/21subsets, 172 matrix, 1.2 zoom, 2.03 mm slice thickness, point spread function correction, 4.0 Gaussian post-reconstruction filtering). Following PET-MR, patients also underwent FDG-PET-CT (128 detector-row Siemens Biograph mCT - Siemens Medical Solutions, Erlangen, Germany) of the torso approximately two hours after injection. Image reconstruction employed CT attenuation correction and iterative reconstruction (24 iterations, 3 subsets, 256 matrix, 1.2 zoom, 1.5 mm slice thickness, time-of-flight, point spread function correction, and no post reconstruction filtering.

### FDG-PET contouring protocol

**Figure 1:**
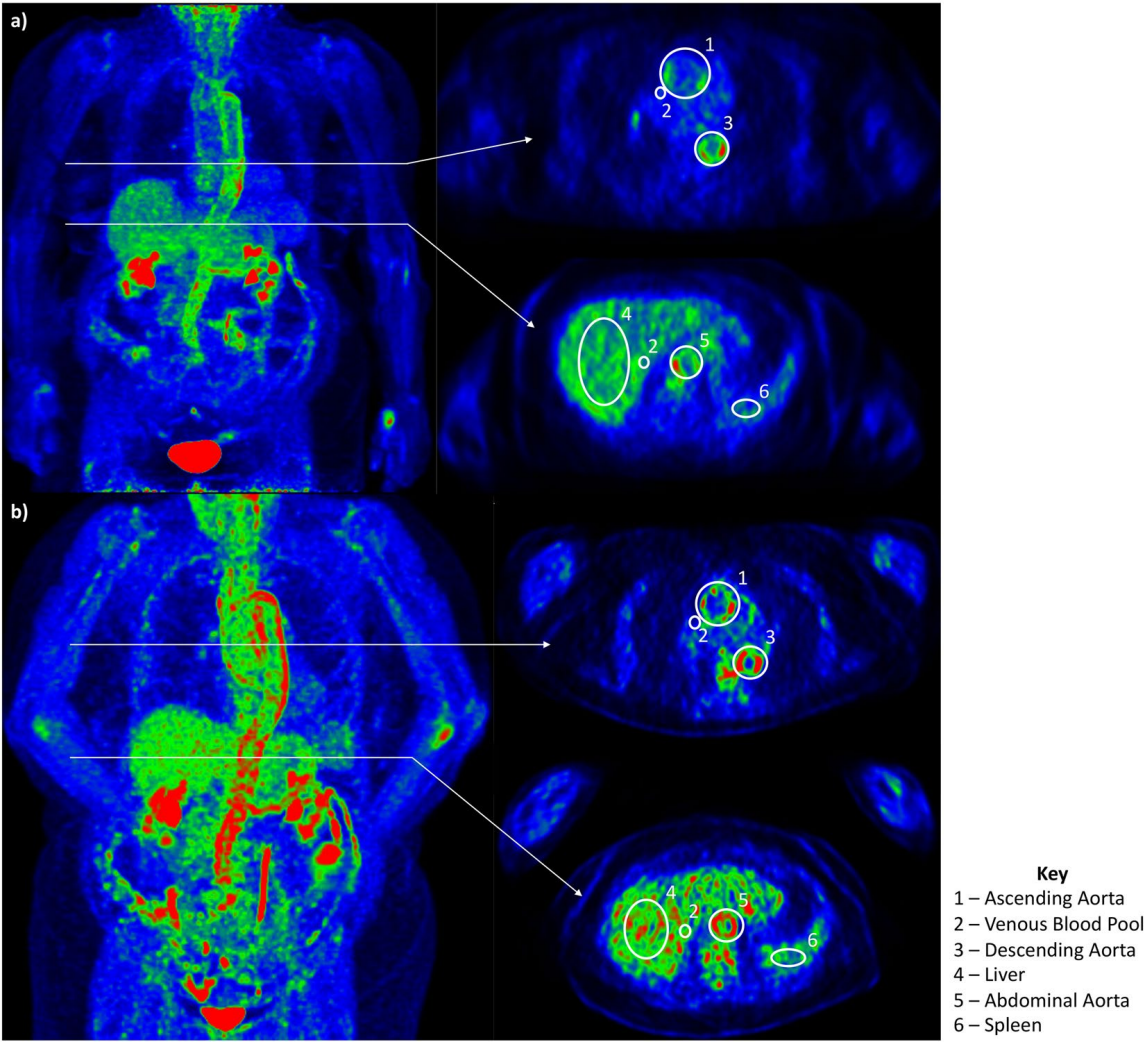
Examples of contoured regions of interest at the one-hour and two-hour imaging time point from FDG-PET studies performed in the same patient. (**a**) One-hour PET 3D reconstructed maximum intensity projection (left) with representative contouring of the ascending^1^ and descending^3^ aorta and venous blood pool^2^ (top), and contouring of the liver^4^, venous blood pool^2^, abdominal aorta^5^, and spleen^6^ (bottom). (**b**) Two-hour PET 3D reconstructed maximum intensity projection (left) with representative contouring of the ascending^1^ and descending^3^ aorta and venous blood pool^2^ (top), and contouring of the liver^4^, venous blood pool^2^, abdominal aorta^5^, and spleen^6^ (bottom). Images windowed to Max 4.0, Min 0.

Arterial FDG uptake was quantified by manually drawing regions of interest (ROI) in OsiriX DICOM Viewer (Version 9.5.2, Bernex, Switzerland), with attention to both CT anatomic location and co-registered PET activity. In an axial view, ROIs that encompassed both the arterial wall and lumen were drawn around five segments of the aorta (ascending aorta, aortic arch, descending thoracic aorta, suprarenal abdominal aorta, infrarenal abdominal aorta), and four branch arteries (right and left common carotid and subclavian arteries) (Fig. 1). This process was repeated for all available image slices and averaged to obtain corresponding maximum standardized uptake values (SUV_Max_) for each arterial region. The mean SUV_Max_ for each of the nine arterial territories were then averaged together to produce a global summary metric of FDG uptake (SUV_Sum_) for the entire aorta and branch arteries of interest.

Average SUV (SUV_Mean_) was also measured in three background reference tissues (liver, blood pool, spleen). Mean liver activity was measured within the right lobe of the liver. Mean venous blood pool activity was evaluated by placing ROIs within the right jugular, superior vena cava, and the inferior vena cava with care to avoid non-venous activity (e.g. physiologic bone marrow and gastrointestinal activity). A volume of interest for mean spleen activity was placed with attention to potential respiratory PET and CT mis-registration artifact and non-splenic activity. Further details of ROI placement can be found in Supplementary Table 1. SUV_Sum_ of the arteries was divided by SUV_Mean_ in background tissue to generate three TBR metrics: TBR_Liver_, TBR_Blood Pool_, and TBR_Spleen_.

### Statistical analyses

One reader contoured all PET images used for subsequent data analysis. A random subset of scans (n = 34, 10%) were contoured a second time to determine intra-rater reliability and were contoured by a second reader to determine interrater-reliability, assessed by Pearson correlation.

Ratios of change between the one-hour and two-hour time points were calculated for tissue-specific FDG distribution. Coefficients of variation (CV, standard deviation/mean values) were calculated for all tissue types at each time point to determine the amount of variability across the study population. An ideal background tissue would have a low coefficient of variation.

Associations between clinical factors and FDG uptake in the arterial wall, background tissues, and arterial wall normalized to background were studied separately at the one and two-hour imaging time points using mixed model multivariable linear regression. Since this was a longitudinal study, patients could undergo the serial delayed imaging acquisition protocol at successive study visits. Mixed model multivariable linear regression was performed using the *lmer* function of the *lme4* package in R with patient identifier as the random effect to account for patients who had multiple study visits. Dependent variables for these models were the global arterial SUV_Sum_, Liver SUV_Mean_, Blood Pool SUV_Mean_, Spleen SUV_Mean_, TBR_Liver_, TBR_Blood Pool_, and TBR_Spleen_. Independent variables included in the mixed models were: 1) demographic variables: age, gender, body mass index (BMI); 2) vasculitis-related variables: total white blood cell count (WBC), c-reactive protein (CRP) (nmol/L), immunosuppressant treatment (yes/no), prednisone daily dose (mg), physician global assessment (PGA), hematocrit; and 3) imaging-related variables: uptake time (in minutes), glomerular filtration rate (GFR)^32^ (mL/min per 1.73 m^2^), and fasting glucose (mmol/L). PGA was scored on a scale of 0 (clinical remission) to 10 (severely active disease). All independent variables were included in each mixed model regression analysis to facilitate comparisons among the different outcome measures. Normalized beta estimates were calculated using the *std beta* function of the *sjstats* package in R to facilitate comparison of effect size across the independent variables within each model. The *Anova* function of the *car* package in R was used to calculate p-values for the models. A p-value < 0.05 was considered statistically significant in all models.

### Ethics approval and consent to participate

All study participants signed written informed consent. The National Institute of Arthritis and Musculoskeletal and Skin Diseases ethics board and the National Institutes of Health Radiation Safety Committee approved the protocol. The research was conducted in accordance with the Declaration of Helsinki.

## Supplementary information

Supplementary Material

## Data Availability

The datasets used and/or analyzed during the current study are available from the corresponding author on request.
