# Supplementary material for: Clinical Factors Associated with Time-Specific Distribution of 18F-Fluorodeoxyglucose in Large-Vessel Vasculitis
Source: Sci Rep. 2019 Oct 23;9:15180. doi: 10.1038/s41598-019-51800-x (PMC6811531; doi:10.1038/s41598-019-51800-x)
Supplement: Supplementary file 1 — Supplementary Material [file 41598_2019_51800_MOESM1_ESM.docx]

**Supplementary Material**

**Clinical Factors Associated with Time-Specific Distribution of 18F-Fluorodeoxyglucose in Large-Vessel Vasculitis**

*Joel S. Rosenblum BS^1^, Kaitlin A. Quinn MD^1,2^, Casey A. Rimland PhD^1,5^, Nehal N. Mehta MD, MSCE, FAHA^3^, Mark A. Ahlman MD^4^, Peter C. Grayson MD, MSc^1^*

*^1^ Systemic Autoimmunity Branch, NIAMS, Bethesda, Maryland, USA; ^2^Division of Rheumatology, MedStar Georgetown University Hospital, Washington, District of Columbia, USA; ^3^University of North Carolina at Chapel Hill School of Medicine, Medical Scientist Training Program, Chapel Hill, NC, USA;  ^4^Cardiovascular Branch, National Heart, Lung, and Blood Institute, Bethesda, Maryland, USA; ^5^ Radiology and Imaging Sciences, National Institutes of Health Clinical Center, Bethesda, Maryland, USA*

**Supplementary Table 1**

Average Number of Contoured Regions of Interest Per Arterial Segment and Background Tissue

| **Tissue** | **Average Number of ROIs**  One-Hour PET | **Average Number of ROIs**  Two-Hour PET |
| --- | --- | --- |
| Ascending Aorta | 22.4 | 40.6 |
| Aortic Arch | 11.4 | 15.1 |
| Descending Thoracic Aorta | 90.5 | 123.8 |
| Suprarenal Aorta | 32.6 | 40.4 |
| Infrarenal Aorta | 20.8 | 44.5 |
| Right Carotid | 23.1 | 68.3 |
| Left Carotid | 23.2 | 68.3 |
| Right Subclavian | 8.4 | 11.7 |
| Left Subclavian | 14.8 | 23.2 |
| Liver | 53.1 | 72.3 |
| Blood Pool | 179.7 | 386.3 |
| Spleen | 25.4 | 36.1 |

**Supplementary Table 2**

Clinical Factors Associated with Blood Pool FDG Uptake at One and Two-Hour Imaging Time Points

|  | **One-Hour** | | | **Two-Hour** | | |
| --- | --- | --- | --- | --- | --- | --- |
| **Variable** | B Estimate | β Estimate | P-value | B Estimate | β Estimate | P-value |
| Sex (female) | -0.08 | 0.16 | 0.09 | 0.10 | 0.21 | 0.02 |
| Age (years) | 0.0006 | 0.06 | 0.64 | 0.005 | 0.41 | <0.01 |
| PGA (0-10) | -0.005 | 0.04 | 0.65 | 0.009 | 0.05 | 0.42 |
| CRP (mg/L) | -0.0005 | 0.05 | 0.61 | -0.0009 | 0.06 | 0.43 |
| Total WBC (10^9^/L) | 0.0001 | 0.05 | 0.94 | 0.007 | 0.1 | 0.22 |
| Fasting Glucose (mg/dL) | 0.001 | 0.09 | 0.23 | 0.001 | 0.09 | 0.28 |
| GFR (mL/min per 1.73 m^2^) | -0.003 | 0.28 | 0.01 | -0.0005 | 0.05 | 0.63 |
| Hematocrit (%) | -0.01 | 0.24 | 0.01 | 0.002 | 0.04 | 0.61 |
| Immune Medications (yes) | 0.02 | 0.03 | 0.59 | -0.004 | 0.09 | 0.22 |
| Prednisone (mg/day) | 0.001 | 0.05 | 0.32 | -0.0007 | 0.03 | 0.63 |
| UT (per minute) | -0.005 | 0.41 | <0.01 | -0.003 | 0.19 | <0.01 |
| BMI | 0.01 | 0.29 | <0.01 | 0.01 | 0.34 | <0.01 |

BMI, Body Mass Index; CRP, C-Reactive Protein; GFR, Glomerular Filtration Rate; Immune Medications (yes), taking immunosuppressant medications other than glucocorticoids; PGA, Physician Global Assessment; Total WBC, Total White Blood Cell Count. Normalized Beta Estimates (β Estimate) given as absolute values; Beta Estimates (B Estimate)

**Supplementary Table 3**

Clinical Factors Associated with Liver FDG Uptake at One and Two-Hour Imaging Time Points

|  | | **One-Hour** | | | | **Two-Hour** | | | |
| --- | --- | --- | --- | --- | --- | --- | --- | --- | --- |
| **Variable** | B Estimate | | β Estimate | P-value | B Estimate | | β Estimate | P-value |  |
| Sex (female) | -0.06 | | 0.07 | 0.46 | 0.15 | | 0.18 | 0.12 |  |
| Age (years) | 0.001 | | 0.09 | 0.50 | 0.006 | | 0.32 | 0.02 |  |
| PGA (0-10) | -0.03 | | 0.15 | 0.10 | 0.02 | | 0.06 | 0.46 |  |
| CRP (mg/L) | 0.0002 | | 0.004 | 0.88 | -0.003 | | 0.12 | 0.17 |  |
| Total WBC (10^9^/L) | 0.0004 | | 0.04 | 0.88 | 0.008 | | 0.08 | 0.44 |  |
| Fasting Glucose (mg/dL) | 0.002 | | 0.1 | 0.27 | 0.0002 | | 0.01 | 0.94 |  |
| GFR (mL/min per 1.73 m^2^) | -0.003 | | 0.22 | 0.06 | -0.0001 | | 0.002 | 0.95 |  |
| Hematocrit (%) | -0.004 | | 0.06 | 0.57 | 0.007 | | 0.08 | 0.46 |  |
| Immune Medications (yes) | 0.08 | | 0.12 | 0.09 | -0.06 | | 0.08 | 0.34 |  |
| Prednisone (mg/day) | -0.0007 | | 0.09 | 0.74 | 0 | | 0.008 | 1.00 |  |
| UT (per minute) | -0.003 | | 0.18 | <0.01 | 0.0002 | | 0.01 | 0.90 |  |
| BMI | 0.02 | | 0.39 | <0.01 | 0.03 | | 0.37 | <0.01 |  |

BMI, Body Mass Index; CRP, C-Reactive Protein; GFR, Glomerular Filtration Rate; Immune Medications (yes), taking immunosuppressant medications other than glucocorticoids; PGA, Physician Global Assessment; Total WBC, Total White Blood Cell Count. Normalized Beta Estimates (β Estimate) given as absolute values; Beta Estimates (B Estimate)

**Supplementary Table 4**

Clinical Factors Associated with Spleen FDG Uptake at One and Two-Hour Imaging Time Points

|  | **One-Hour** | | | **Two-Hour** | | |
| --- | --- | --- | --- | --- | --- | --- |
| **Variable** | B Estimate | β Estimate | P-value | B Estimate | β Estimate | P-value |
| Sex (female) | 0.03 | 0.03 | 0.70 | 0.13 | 0.12 | 0.39 |
| Age (years) | 0.001 | 0.14 | 0.53 | 0.006 | 0.24 | 0.15 |
| PGA (0-10) | -0.03 | 0.17 | 0.07 | 0.02 | 0.06 | 0.52 |
| CRP (mg/L) | 0.002 | 0.1 | 0.07 | 0.001 | 0.05 | 0.56 |
| Total WBC (10^9^/L) | -0.0007 | 0.13 | 0.74 | 0.02 | 0.16 | 0.11 |
| Fasting Glucose (mg/dL) | 0.0 | 0.05 | 0.96 | -0.0007 | 0.02 | 0.80 |
| GFR (mL/min per 1.73 m^2^) | -0.002 | 0.12 | 0.26 | 0.001 | 0.05 | 0.73 |
| Hematocrit (%) | -0.006 | 0.14 | 0.40 | -0.008 | 0.07 | 0.53 |
| Immune Medications (yes) | -0.01 | 0.02 | 0.80 | -0.08 | 0.09 | 0.24 |
| Prednisone (mg/day) | 0.002 | 0.03 | 0.18 | -0.003 | 0.08 | 0.38 |
| UT (per minute) | 0.001 | 0.08 | 0.24 | -0.0008 | 0.03 | 0.63 |
| BMI | 0.02 | 0.44 | <0.01 | 0.05 | 0.49 | <0.01 |

BMI, Body Mass Index; CRP, C-Reactive Protein; GFR, Glomerular Filtration Rate; Immune Medications (yes), taking immunosuppressant medications other than glucocorticoids; PGA, Physician Global Assessment; Total WBC, Total White Blood Cell Count. Normalized Beta Estimates (β Estimate) given as absolute values; Beta Estimates (B Estimate).

**Supplementary Table 5**

Factors Associated with Arterial Uptake Normalized to Blood Pool at One and Two-Hour Time Points

|  | **One-Hour** | | | **Two-Hour** | | |
| --- | --- | --- | --- | --- | --- | --- |
| **Variable** | B Estimate | β Estimate | P-value | B Estimate | β Estimate | P-value |
| Sex (female) | -0.02 | 0.01 | 0.85 | -0.14 | 0.08 | 0.50 |
| Age (years) | 0.007 | 0.38 | <0.01 | 0.01 | 0.25 | 0.07 |
| PGA (0-10) | -0.005 | 0.02 | 0.79 | -0.02 | 0.04 | 0.69 |
| CRP (mg/L) | 0.004 | 0.32 | 0.03 | 0.02 | 0.33 | <0.01 |
| Total WBC (10^9^/L) | 0.0003 | 0.1 | 0.91 | -0.02 | 0.07 | 0.51 |
| Fasting Glucose (mg/dL) | -0.003 | 0.18 | 0.12 | -0.01 | 0.2 | 0.02 |
| GFR (mL/min per 1.73 m^2^) | 0.004 | 0.25 | 0.04 | 0.005 | 0.16 | 0.21 |
| Hematocrit (%) | 0.002 | 0.07 | 0.79 | -0.01 | 0.06 | 0.54 |
| Immune Medications (yes) | 0.01 | 0.1 | 0.81 | -0.22 | 0.13 | 0.10 |
| Prednisone (mg/day) | 0.002 | 0.02 | 0.24 | 0.004 | 0.07 | 0.48 |
| UT (per minute) | 0.005 | 0.32 | <0.01 | 0.006 | 0.11 | 0.08 |
| BMI | 0.008 | 0.15 | 0.25 | 0.02 | 0.11 | 0.31 |

BMI, Body Mass Index; CRP, C-Reactive Protein; GFR, Glomerular Filtration Rate; Immune Medications (yes), taking immunosuppressant medications other than glucocorticoids; PGA, Physician Global Assessment; Total WBC, Total White Blood Cell Count. Normalized Beta Estimates (β Estimate) given as absolute values; Beta Estimates (B Estimate)

**Supplementary Table 6**

Factors Associated with Arterial Uptake Normalized to Liver at One and Two-hour Imaging Time Points

|  | **One-Hour** | | | | **Two-Hour** | | | |  |
| --- | --- | --- | --- | --- | --- | --- | --- | --- | --- |
| **Variable** | | B Estimate | β Estimate | P-value | | B Estimate | β Estimate | P-value | |
| Sex (female) | | -0.007 | 0.02 | 0.89 | | -0.04 | 0.04 | 0.72 | |
| Age (years) | | 0.004 | 0.4 | <0.01 | | 0.008 | 0.34 | 0.02 | |
| PGA (0-10) | | 0.01 | 0.09 | 0.30 | | -0.005 | 0.02 | 0.82 | |
| CRP (mg/L) | | 0.004 | 0.33 | <0.01 | | 0.01 | 0.36 | <0.01 | |
| Total WBC (10^9^/L) | | -0.007 | 0.1 | 0.65 | | -0.005 | 0.04 | 0.68 | |
| Fasting Glucose (mg/dL) | | -0.002 | 0.18 | 0.07 | | -0.005 | 0.17 | 0.046 | |
| GFR (mL/min per 1.73 m^2^) | | 0.002 | 0.2 | 0.08 | | 0.003 | 0.14 | 0.29 | |
| Hematocrit (%) | | 0.002 | 0.03 | 0.65 | | -0.006 | 0.05 | 0.60 | |
| Immune Medications (yes) | | -0.07 | 0.16 | 0.03 | | 0.11 | 0.12 | 0.11 | |
| Prednisone (mg/day) | | -0.0006 | 0 | 0.60 | | 0.0005 | 0.02 | 0.86 | |
| UT (per minute) | | 0.001 | 0.11 | 0.08 | | 0.0005 | 0.02 | 0.76 | |
| BMI | | 0.004 | 0.1 | 0.36 | | 0.008 | 0.09 | 0.40 | |

BMI, Body Mass Index; CRP, C-Reactive Protein; GFR, Glomerular Filtration Rate; Immune Medications (yes), taking immunosuppressant medications other than glucocorticoids; PGA, Physician Global Assessment; Total WBC, Total White Blood Cell Count. Normalized Beta Estimates (β Estimate) given as absolute values; Beta Estimates (B Estimate)

**Supplementary Table 7**

Factors Associated with Arterial Uptake Normalized to Spleen at One and Two-Hour Imaging Time Points

|  | **One-Hour** | | | **Two-Hour** | | |
| --- | --- | --- | --- | --- | --- | --- |
| **Variable** | B Estimate | β Estimate | P-value | B Estimate | β Estimate | P-value |
| Sex (female) | -0.05 | 0.07 | 0.44 | -0.01 | 0.007 | 0.95 |
| Age (years) | 0.006 | 0.38 | <0.01 | 0.009 | 0.36 | 0.02 |
| PGA (0-10) | 0.02 | 0.13 | 0.20 | 0.02 | 0.06 | 0.59 |
| CRP (mg/L) | 0.003 | 0.24 | 0.045 | 0.005 | 0.18 | 0.05 |
| Total WBC (10^9^/L) | 0.0001 | 0.17 | 0.94 | -0.01 | 0.06 | 0.55 |
| Fasting Glucose (mg/dL) | -0.0009 | 0.13 | 0.57 | -0.004 | 0.12 | 0.18 |
| GFR (mL/min per 1.73 m^2^) | 0.002 | 0.15 | 0.17 | 0.003 | 0.13 | 0.35 |
| Hematocrit (%) | -0.002 | 0.02 | 0.78 | -0.002 | 0.01 | 0.88 |
| Immune Medications (yes) | -0.04 | 0.05 | 0.41 | -0.09 | 0.09 | 0.27 |
| Prednisone (mg/day) | -0.001 | 0.04 | 0.50 | 0.0002 | 0.008 | 0.95 |
| UT (per minute) | -0.0006 | 0.04 | 0.60 | 0.0007 | 0.02 | 0.75 |
| BMI | 0.002 | 0.06 | 0.67 | -0.0003 | 0.001 | 0.98 |

BMI, Body Mass Index; CRP, C-Reactive Protein; GFR, Glomerular Filtration Rate; Immune Medications (yes), taking immunosuppressant medications other than glucocorticoids; PGA, Physician Global Assessment; Total WBC, Total White Blood Cell Count. Normalized Beta Estimates (β Estimate) given as absolute values; Beta Estimates (B Estimate)

**Supplementary Figure 1**

Intra-reader correlation of image contouring for all tissues

a) Intra-Reader correlation of SUV_sum_ Artery b) Intra-Reader correlation of SUV_mean_ Blood Pool c) Intra-Reader correlation of SUV_mean_ Liver d) Intra-Reader correlation of SUV_mean_ Spleen

**Supplementary Figure 2**

Inter-reader correlation of contouring for all tissues

a) Inter-Reader correlation of SUV_sum_ Artery b) Inter-Reader correlation of SUV_mean_ Blood Pool c) Inter-Reader Correlation of SUV_mean_ Liver d) Inter-Reader Correlation of SUV_mean_ Spleen
